# Modified Unipolar Hemiarthroplasty for the Treatment of Metastatic Lesions of Proximal Femur with Pathological Fractures: Case Series of Six Patients

**DOI:** 10.5704/MOJ.2003.018

**Published:** 2020-03

**Authors:** RY Kow, KL Goh

**Affiliations:** 1 Department of Orthopaedic, Hospital Tengku Ampuan Afzan (HTAA), Kuantan, Malaysia; 2 Department of Orthopaedic, International Islamic, University Malaysia (IIUM), Kuantan, Malaysia

Dear editor,

We read with great interest the case series by Lim *et al* in the November 2019 issue entitled “Modified Unipolar Hemiarthroplasty for the Treatment of Metastatic Lesions of Proximal Femur with Pathological Fractures: Case Series of Six Patients”^1^. In their paper, they presented their experience in using “modified unipolar hemiarthroplasty for reconstruction after proximal femur resection for pathological fracture of the proximal femur secondary to metastatic lesions. This innovative, cost-effective implant can replace the otherwise expensive endoprosthesis.

The aim of treating patients with pathological fracture of the proximal femur secondary to metastatic lesion is to alleviate pain, improve function and quality of life and to prevent further surgery^1-3^. Surgical options include reconstruction with hemiarthroplasty and stabilisation with cephalomedullary nailing with/without cement augmentation. The factors to consider when considering the surgical option include site of fracture (neck, intertrochanteric or subtrochanteric), the patient’s prognosis (predicted survival time) and patient’s suitability for surgery. In the case series presented by the authors, all patients had poor prognosis. All except one patient had modified Baur score of 0-1, indicating a median overall survival of 4.8 months. On top of that, all patients had at least two of the negative prognostic factors (pathological fracture, visceral metastasis, lung cancer and anemia) based on the Scandinavian Sarcoma Group skeletal metastasis register, implicating an extremely short survival. All but one patient deceased within four months after the surgery. Besides that, two of the patients had other long bone lesions (one tibia and one femur) that may complicate the healing and ambulatory status of the patients.

Taking into consideration of all these factors, we are of opinion that those patients will be benefitted from “minimal surgery” of long cephalomedullary nail and cement augmentation. Through this procedure, the surgery time will be shortened, and the blood loss will be minimised. Bearing in mind that all patients had lung metastasis at the time of surgery, a prolonged surgery will increase the risk of general anaesthesia (if operated under general anaesthesia). After this procedure, the patient will be able to ambulate immediately with wheelchair, hence the complications of immobilisation will be minimised. Meta-analysis by Putnam *et al* show that re-operation rates is the same between intramedullary nailing and arthroplasty in treating pathological fracture of proximal femur pertrochanteric region^3^. Finally, the cost of a cephalomedullary nail do not differ much compare to the implant proposed by the authors. While the authors’ innovative implant is commendable, it should be used in carefully selected patients so that the benefits outweigh the risk of the procedure.
