# Supplementary material for: Modified Unipolar Hemiarthroplasty for the Treatment of Metastatic Lesions of Proximal Femur with Pathological Fractures: Case Series of Six Patients
Source: Malays Orthop J. 2020 Mar;14(1):98–9. doi: 10.5704/MOJ.2003.018 (PMC7156173; doi:10.5704/MOJ.2003.018)
Supplement: Related texts

# Reply: Response to Letter to Editor

**DOI:** 10.5704/MOJ.1911.004

**Published:** 2019-09

**Authors:** Mohammad Anwar Hau Abdullah

Thank you very much for the interest shown in our paper and also the very valuable comment and opinion given. It is highly appreciated.

As mentioned by Issack *et al*^1^, pathologic femoral neck fractures secondary to metastatic disease are best treated with prosthetic replacement. They also mentioned that the indications for choosing one surgical implant option over another are not clear for intertrochanteric and subtrochanteric fractures1. We do agree with the opinion of long cephalomedullary nail and cement augmentation if there is adequate bone in the femoral neck and head to support the implant.

However, due to extensive metastatic lesions, not every case of proximal femoral metastasis with pathological fracture will have adequate bone stock to support cephalomedullary nail in spite of cement augmentation. We experienced construction failure in some earlier cases, which were stabilised with nail or plate and augmented with cement, secondary to disease progression. For the patients in our report we thought they were also at high risk of early failure with the similar methods of fixation. Thus, we embarked on the technique of “modified unipolar hemiarthroplasty”.

It was the beginning of our learning curve while those cases were performed, we have learned a lot, and since then, were more selective, the surgical time and blood lost had also much reduced. In carefully selected patients, we are constantly striving to provide much better quality of life with lesser construct failure with our modified unipolar hemiarthroplasty.

Once again, we appreciated and thank you very much for you interest in our paper.

## References

[1] Issack PS, Barker J, Baker M, Kotwal SY, Lane JM (2014). Surgical Management of Metastatic Disease of the Proximal Part of the Femur.. J Bone Joint Surg Am..
